# Sperm donor attitudes and experiences with direct-to-consumer genetic testing

**DOI:** 10.1016/j.xfre.2022.12.004

**Published:** 2023-02-24

**Authors:** Sascha Wodoslawsky, Joy Fatunbi, Rebecca Mercier, Andrea Mechanick Braverman

**Affiliations:** a Sidney Kimmel Medical College at Thomas Jefferson University, Philadelphia, Pennsylvania; b Department of Obstetrics and Gynecology, Sidney Kimmel Medical College at Thomas Jefferson University, Philadelphia, Pennsylvania

**Keywords:** Donor conception, sperm banks, spermatozoa, direct-to-consumer screening and testing, confidentiality

## Abstract

**Objective:**

To identify factors influencing sperm donor willingness to participate in direct-to-consumer genetic testing, comfort with sharing genetically identifiable data in commercial genetic testing databases, and likelihood to donate sperm again.

**Design:**

Cross-sectional online anonymous survey.

**Setting:**

Multicenter, 2 large American sperm banks from July 1, 2020 to July10, 2021.

**Patient(s):**

Sperm donors from 1980 to 2020.

**Intervention(s):**

None.

**Main outcome measure(s):**

Associations between donor demographic characteristics, donation history, and attitudes toward direct-to-consumer genetic testing.

**Result(s):**

A total of 396 donors completed the survey. Most donations (61.5%) occurred from 2010 to 2020, and 34.3% were nonidentified donations. Nonidentified donors were less comfortable with their genetic data being shared than open-identity donors (25.4% vs. 43.8%) and were less likely than open-identity donors to donate sperm again (43.3% vs. 72.1%). Donors who donated after the inception of direct-to-consumer genetic testing in 2007 were less likely to participate in commercial genetic testing than those who donated before 2007 (25.8% vs. 37.1%). Most donors (87.4%) have disclosed their donation(s) to current partners, but fewer have disclosed them to their families (56.6%) or children (30.5%). Of the donors who had been contacted by donor-conceived persons, 79.5% were identified via direct-to-consumer genetic testing. Overall, 61.1% of donors would donate again regardless of direct-to-consumer genetic testing.

**Conclusion(s):**

Direct-to-consumer genetic testing is playing a dynamic role in sperm donor identification, but donors seem willing to donate again. Implication counseling regarding future linkage and contact from donor-conceived persons needs to be standardized for potential donors before donation.

Sperm donation and the use of sperm banks in assisted reproduction is a valuable resource for family building in azoospermic heterosexual couples, couples without a male or with a transmale partner, and single mothers by choice (1). Regulations for gamete donation and the identification of donors vary widely worldwide. Sperm donation was traditionally anonymous or nonidentified, meaning the identifying factors of those involved in the donation process, including donors, recipients, and medical or coordinating professionals, would not be shared between the donor, the recipient, or any persons conceived from the donation (2). Open-identity donations are increasing in the United States and worldwide, and many donor-conceived persons (DCP) are advocating for their right to claim identifying donor information once they reach majority age (3, 4, 5, 6). At least 14 countries have adopted laws and regulations requiring that donor identifying information be made available to DCP at majority age (7, 8).

Open-identity sperm donation in the United States has been offered to donors as early as 1982 (9). However, only a minority of states, like Washington, Colorado, and California, have legislation in place regarding donor identity disclosure (10, 11, 12). Open-identity donations are increasing in the United States, but nonidentified donations continue to be made although anonymity can no longer be assured (4, 6, 13, 14).

Accessibility to commercial genetic testing from companies like 23andMe and Ancestry has drastically changed since it was first launched in 2007 (15). By 2019, over 26 million people have reportedly participated in direct-to-consumer genetic testing (DTC-GT) worldwide (16, 17). These companies offer convenient testing kits to consumers looking to trace genealogical ancestry or to gain insight into inherited health conditions, and consumers can use these company databases to connect with genetically related persons (4, 18). These kits are distinct from the expanded carrier screening offered to donors by sperm banks. Expanded carrier screening is ordered by a health care provider, and all identifying factors are kept confidential between the provider, the donor, and the genetic counselors involved (15).

Increasing accounts from donors and DCP around the world are emerging regarding the ease of identification through DTC-GT and social media accounts, but limited research has fully examined the scope and repercussions of this reality (2, 19, 20, 21). A major transition to open-identity has begun as the use of donor sperm is becoming a less secretive or even shameful practice (4). Since the commercialization of DTC-GT, donors are more aware that personal information can easily be shared online and there has been a shift toward greater psychoeducational counseling from mental health professionals before their donations compared with pre-2007 donors (1, 19, 22, 23). However, limited studies have explored the collective willingness of donors to release identifiable information to DCP and their families now that DTC-GT exists (4, 8, 20, 21, 24).

Direct-to-consumer genetic testing and their databases pose a unique challenge for sperm banks and the many nonidentified donors who did not foresee such a resource existing at the time of their donation. An analysis of the perspectives of past and current sperm donors on DTC-GT is warranted to understand how this service is affecting sperm donation in the United States and what this new reality could mean for American sperm banks in the future. The objective of this study was to assess the factors that influence sperm donor willingness to participate in DTC-GT, comfort with DTC-GT databases sharing genetically identifiable data, and likelihood to donate sperm again.

## Materials and methods

Sperm donors who made at least 1 sperm donation between January 1980 and January 2020 at Fairfax Cryobank (FCB) or California Cryobank (CCB) were contacted via email. Donors were invited to complete an anonymous retrospective online survey via a link included in the participant recruitment email. Donors from these 2 sperm banks are routinely contacted via email outside the scope of this study for marketing, recruitment, or other sperm bank communication. Sperm donor contact lists were confidentially managed by the respective sperm banks. No identifiable information was collected from the participants. Participation was completely anonymous and voluntary. Donors were not compensated for their participation in this study. Data on sperm donors with outdated or undeliverable emails were not collected.

The anonymous survey was created on the Qualtrics platform and consisted of 39 questions (Supplemental Material [available online]). A link to the survey was distributed via email, leading to an introductory page that included background information about the study and definitions for the terms direct-to-consumer genetic testing (i.e., “At home DNA testing kits like 23andMe, Ancestry, etc.”), donor-conceived persons/offspring (i.e., “Children conceived using your donated sperm sample”), nonidentified donor (i.e., “No option of contact or future information exchange”), open-identity donor (i.e., “Agreed to participate in at least one facilitated contact with offspring once they are age 18”), identification disclosure donor (i.e., “Sperm bank to provide my identifying information to offspring once they are age 18”). The survey was available online from July 1, 2020, to July 10, 2021. The questions were not piloted by donors before survey distribution, but each survey question was constructed and reviewed for relevance and wording by several experts in the field of gamete donation, including medical and mental health professionals. The answer format varied and included single answer, multiple answer, Likert scale, and full text box options.

Survey responses were collected, and data were aggregated through the Qualtrics platform. The following information was collected: donor demographic characteristics, including race, religion, and the highest level of education; donation information, including age at first donation, year of donation(s), donor disclosure type (i.e., nonidentified, open-identity, or identification disclosure), and the original motivation for donation (i.e., altruism, financial compensation, personal satisfaction, and/or health testing); family and disclosure information, including partner status and number of children at the time of donation, current partner status and number of children, and disclosure of donor status to current partner, family, and children; DCP contact preferences and experiences, including willingness to be contacted by DCP (i.e., “Are you open to contact with your donor-conceived offspring?”), experience if contacted, and method of identification by DCP; DTC-GT experience, including participation in DTC-GT, timing of DTC-GT participation, reasons for DTC-GT participation or refusing participation, and family member participation in DTC-GT; DTC-GT opinions, including acceptance of and comfort level with genetic data sharing by DTC-GT companies (i.e., “As a sperm donor, how comfortable are you that companies such as 23andMe and Ancestry are sharing the identity of genetically related persons?”), likelihood of subsequent sperm donation (i.e., “Would you feel comfortable donating sperm again knowing that companies such as 23andMe and Ancestry are sharing the identity of genetically related persons?”), and a free text box for additional commentary.

### Statistical Analysis

The outcome of attitudes toward DTC-GT was defined by 3 variables: self-report of participation in DTC-GT (yes or no), comfort with DTC-GT companies sharing genetic data (5-item Likert scale combined to a 3-level scale for analysis), and likelihood to donate again (yes, no, or unsure). The type of donation was collected as a 3-level variable (nonidentified, open-identity, or identification disclosure), of which open-identity and identification disclosure categories were combined and defined as “open” for analysis. The distinction between open-identity and identification disclosure was originally made in the survey to accommodate the language at 2 different banks; open-identity donors agree to at least 1 communication with DCP at majority age, whereas identification disclosure donors allow banks to release any identifying information to DCP at majority age. The motivation for donation was a multilevel variable (Table 1) where participants were allowed to select multiple motivations. Subjects reported all years of donation, and these were both combined into decades and dichotomized to pre- and post-2007 (i.e., pre and post DTC-GT).

**Table 1:** Donor cohort information.

Demographic characteristics	
Age at first donation (y)	26.5 ± 5.4
Race	
Caucasian	335 (84.6)
Black or African American	11 (2.8)
Asian	14 (3.5)
Hispanic	19 (4.8)
Other	17 (4.3)
Religion	
Nonreligious	210 (53.0)
Christian	85 (21.5)
Jewish	12 (3.0)
Muslim	2 (0.5)
Other	87 (22.0)
Education	
Undergraduate	182 (46.0)
Graduate	114 (28.8)
Professional	80 (20.2)
Other	20 (5.0)
Donation history	

Year of donation	
Before 2007	97 (24.5)
After 2007	299 (75.5)
Donor type	
Nonidentified	134 (33.8)
Open-identity or ID disclosure	251 (63.4)
Unsure	11 (2.8)
Original motivation for donation^a^	
Altruism	255 (64.4)
Financial compensation	295 (74.5)
Personal satisfaction	85 (21.5)
Health testing	79 (19.9)
Family status and disclosure	

Partner	
Having a partner at donation	189 (47.7)
Having a partner currently	301 (76.0)
Disclosed to current partner	263 (87.4)
Children	
Have children currently	162 (40.9)
Disclosed to children	51 (31.5)
Family	
Disclosed to family	224 (56.6)

*Note:* Age presented as mean ± SD. Data presented as n (%). ID = identification.

a Respondents could choose multiple motivations for donation.

Statistical analysis was performed using SPSS 27 (IBM Corp, Armonk, NY). Descriptive statistics were used to summarize qualitative variables. Means of continuous variables were compared with *t* test or ANOVA testing for multilevel variables. Chi-square tests were used to assess associations between subject characteristics and the study outcomes. Responses with >50% of relevant data missing were excluded from the analysis.

### Ethical Approval

The study and final survey were approved by the Thomas Jefferson University institutional review board.

## Results

### Survey Response

At CCB, a pool of 1,797 retired donors was considered for the study, of which 1,356 were excluded because of a history of unresponsiveness, donor indication that they no longer wished to be contacted, or lack of email address. A total of 441 donors were contacted at CCB, and 31 emails were returned as undeliverable (n = 410). At FCB, 1,865 donors were contacted, of which 101 emails were undeliverable (n = 1,764). Details on the number of donors who did not wish to be contacted were unavailable at FCB. A total of 2,174 email invitations were sent, and 396 donors responded to the survey. The survey response rate was 97 of 410 (23.7%) from CCB donors and 284 of 1,764 (16.1%) from FCB donors. The remaining 15 respondents did not recall which bank they used for their donation. The overall response rate for the delivered survey was 18.2% (396/2,174).

Of the 396 respondents, 329 (83.1%) donors made multiple donations over several years for a reported total of 1,031 donations in all. The earliest year of donation included in this analysis was reported to be 1988. The number of offspring that resulted from these donations could not be reported as donors are not kept informed of such data. Data on sperm donors with outdated or undeliverable emails were not collected; therefore, limited demographic information is known about these donors.

### Donor Cohort Demographic Characteristics and Donation History

The mean age at first donation was 26.5 ± 5.4 years (median 25.0 years, range 18–42 years). Most donors in this cohort self-identified as Caucasian (335/396, 84.6%) and nonreligious (210/396, 53.0%). The highest education levels most reported were undergraduate (182/396, 46.0%) and graduate (114/396, 28.8%) degrees. Most donations in this cohort (634/1,031, 61.5%) occurred between 2010 and 2020, with 72.4% (746/1,031) of donations occurring after the inception of the first DTC-GT service in 2007. Over half of the donors (215/396, 54.3%) reported multiple motivations for their donation (Table 1).

Only 113 of 396 (28.5%) donors reported participating in DTC-GT, 13 (11.5%) of which reported that one of their motivations for DTC-GT participation was to aid in making themselves identifiable to DCP (Table 2). Other reasons for DTC-GT participation were to obtain ancestry information (86/113, 76.1%) or out of general curiosity (68/113, 60.2%). Of the 283 of 396 (71.5%) donors who have not done DTC-GT, 49 (17.3%) reported that they refrained from participating in the service to maintain their anonymity from potential DCP. Although most donors (352/396, 88.9%) were not contacted by DCP, those who were contacted were mainly identified via DTC-GT databases (35/44, 79.5%). Comfort levels with genetic data sharing within DTC-GT databases were reported; 37.1% (147/396) of all respondents stated that they felt comfortable, 30.3% (120/396) were neutral, and 32.6% (129/396) felt uncomfortable (Table 3). Most donors (242/396, 61.1%) stated that they would donate sperm again even though identification through DTC-GT is possible (Table 4).

**Table 2:** Characteristics influencing attitudes toward participation in DTC-GT.

Characteristic	Participation in DTC-GT		*P* value
	Yes N *=* 113	No N = 283	
Year of donation			.031^a^
Before 2007	36 (37.1)	61 (62.9)	
After 2007	77 (25.8)	222 (74.2)	
Type of donation			.520
Nonidentified	41 (30.6)	93 (69.4)	
Open-identity or ID disclosure	69 (27.5)	182 (72.5)	
Original motivation for donation			
Altruism	73 (28.6)	182 (71.4)	.956
Financial compensation	81 (27.5)	214 (72.5)	.417
Personal satisfaction	25 (29.4)	60 (70.6)	.840
Health testing	28 (35.4)	51 (64.6)	.129

*Note:* Data presented as n (%). DTC-GT= direct-to-consumer genetic testing; ID = identification.

a Statistically significant result (*P* < .05).

**Table 3:** Characteristics affecting comfort with genetic data sharing by DTC-GT companies.

Characteristic	Comfort with genetic data sharing			*P* value
	Comfortable N = 147	Neutral N = 120	Uncomfortable N = 129	
Year of donation				.247
Before 2007	38 (39.2)	23 (23.7)	36 (37.1)	
After 2007	109 (36.5)	97 (32.4)	93 (31.1)	
Type of donation				.000^a^
Nonidentified	34 (25.4)	37 (27.6)	63 (47.0)	
Open-identity or ID disclosure	110 (43.8)	80 (31.9)	61 (24.3)	
Original motivation for donation				
Altruism	100 (39.2)	73 (28.6)	82 (32.2)	.461
Financial compensation	107 (36.3)	93 (31.5)	95 (32.2)	.656
Personal satisfaction	31 (36.5)	27 (31.8)	27 (31.8)	.946
Health testing	31 (39.2)	22 (27.8)	26 (32.9)	.853
Family status and disclosure				
Partnered at donation	78 (41.3)	64 (33.9)	47 (24.9)	.015^a^
Partnered currently	110 (36.5)	91 (30.2)	100 (33.2)	.871
Disclosed to current partner	101 (38.4)	79 (30.0)	83 (31.6)	.428
Have children now	60 (37.0)	54 (33.3)	48 (29.6)	.690

*Note*: Data presented as n (%). DTC-GT = direct-to-consumer genetic testing; ID = identification.

a Statistically significant result (*P* < .05).

**Table 4:** Characteristics affecting donor likelihood to donate again.

Characteristic	Likelihood to donate again			*P* value
	Yes N = 242	Unsure N = 91	No N = 63	
Year of donation				.067
Before 2007	51 (52.6)	24 (24.7)	22 (22.7)	
After 2007	191 (63.9)	67 (22.4)	41(13.7)	
Type of donation				.000^a^
Nonidentified	58 (43.3)	37 (27.6)	39 (29.1)	
Open-identity or ID disclosure	181 (72.1)	48 (19.1)	22 (8.8)	
Original motivation for donation				
Altruism	160 (73.0)	26 (11.9)	33 (15.1)	.007^a^
Financial compensation	180 (71.4)	24 (9.5)	48 (19.1)	.936
Personal satisfaction	60 (70.6)	13 (15.3)	12 (14.1)	.101
Health testing	45 (56.9)	24 (30.4)	10 (12.7)	.193
Family status and disclosure				
Partnered at donation	119 (63.0)	44 (23.3)	26 (13.8)	.564
Disclosed to current partner	168 (63.9)	59 (22.4)	36 (13.7)	.000^a^
Not disclosed to current partner	13 (37.1)	9 (25.7)	13 (37.1)	.000^a^

*Note:* Data presented as n (%). ID = identification.

a Statistically significant result (*P* < .05).

Donors who made their donation after the inception of the first DTC-GT service in 2007 were less likely to participate in DTC-GT than those who donated before 2007 (25.8% vs. 37.1%, *P* = .031) (Table 2). Donation before or after 2007 was not significantly associated with the donor's comfort level of DTC-GT companies sharing genetically identifiable information in their databases, nor did it statistically affect their likelihood of donating sperm again (Tables 3 and 4).

Nonidentified donors were less comfortable with genetic data sharing than open-identity donors (25.4% vs. 43.8%, *P* < .01) (Table 3). Nonidentified donors were also less likely than open-identity donors to donate sperm again (43.3% vs. 72.1%, *P* < .01) (Table 4). The type of donation did not affect donor participation in DTC-GT in a significant manner. The 113 donors who participated in DTC-GT were more likely to donate again than the 283 donors who had not done DTC-GT (69.9% vs. 57.6%, *P* = .028).

Motivators for donation did not significantly affect donor attitudes toward DTC-GT. The 255 donors who reported altruism as one of their motivators for donation were slightly more likely to donate again than those who did not donate for altruistic purposes (62.7% vs. 58.2%, *P* = .007) (Table 4), but motivation was not significantly associated with participation in DTC-GT or comfort with genetic data sharing.

The 189 donors who reported having a partner at the time of their donation were more comfortable with genetic data sharing than those who did not have a partner (41.3% vs. 35.3%, *P* = .015) (Table 3). Having a partner at the time of donation did not significantly affect the likelihood of donating sperm again (*P* = .564). Most donors (224/396, 56.6%) disclosed their donor status to family members. Of the 301 donors who reported being currently partnered, 263 (87.4%) disclosed their donation to their partners. Of the 162 donors who reported currently having children, 51 (31.5%) disclosed to their children (Table 1). Currently having a partner or children did not significantly affect donor comfort with genetic data sharing or donor openness to contact from DCP (Table 3). The 263 donors who disclosed their donor status to their current partners were more likely to donate sperm again than those who had not disclosed it to their current partners (63.9% vs. 37.1%, *P* < .01) (Table 4).

## Discussion

Our data shows that DTC-GT companies and their genetic databases are playing a role in the identification of sperm donors by DCP and their families. Several studies have addressed the concern that nonidentified donations are becoming increasingly obsolete with advancing genetic technologies (18, 25, 26). Our results indicate that this is indeed a reality, as 79.5% of the donors who have been contacted by DCP stated they were identified through DTC-GT databases. Thus, it is important to take the realities of DTC-GT into consideration and to revise the current gamete donation practices in the United States.

Our study found that 28.5% of donors reported participating in DTC-GT, and many of the respondents who had not participated in DTC-GT said they intended to do so soon. Current data on the adoption of DTC-GT in the general United States population or populations with medical conditions is limited (23). An early study from 2008 concluded that 22% of respondents knew about DTC-GT, but only 0.3% had participated (27), whereas another study in 2009 saw an increase in the numbers when surveying Facebook users; 47% were aware of DTC-GT, whereas 6% had participated (22, 28). Direct-to-consumer genetic testing has continued to grow in popularity with increased media coverage and decreased cost. Over 26 million people have participated in DTC-GT worldwide as of 2019 (16). A recent survey of over 10,000 people in the United States in 2019 concluded that 21.8% of respondents had received genetic testing, of which 76.6% of those tests were performed through DTC-GT kits as opposed to clinical testing ordered by a physician or genetic counselor (29).

Despite the increasing popularity of DTC-GT, donors may not completely understand the impact that some of these advancing technologies may have on their wish for anonymity or no future contact. The disclosure patterns in our study highlighted this point, as just over half of the subjects disclosed their donor status to family members, and only a third disclosed it to their children. Although it is understandable that their children may not yet be of age, it is important to consider that DTC-GT databases connect both immediate and extended genetic matches. Thus, any family member of a sperm donor who participates in DTC-GT can inadvertently discover a donor’s status if linked to a genetically related person. These databases are accessible to DCP looking for their donor or donor siblings, and vice versa, violating the rights of recipients and donors established by anonymity or controlled contact (30). Our findings show that donors who made their donation after the inception of the first DTC-GT service in 2007 were less likely to participate in DTC-GT. This is a possible artifact of the growing awareness and prompted reflections on the consequences of donation through adequate counseling. Thus, this generation of donors is not necessarily more comfortable with information sharing; they are simply better informed.

Over the last few decades, there has been a global trend toward laws and regulations that give DCP the right to ask for donor identifying information at majority age (4, 7, 13). These changes are in response to both DCP desire to connect with their donors and to the legislation clarifying the social responsibility or ethical obligation of donors to disclose inherited genetic conditions or family histories (7, 31, 32). For example, Colorado recently passed a law eliminating donor anonymity (11). One of the primary concerns with removing nonidentified donation is the belief that donor numbers will decrease, leading to a donor shortage (18). In theory, these concerns are valid. A study by Cohen et al. (14) in 2016 stated that in a representative sample of American donors, 29% of nonidentified sperm donors would refuse to donate if the law required them to release any identifying information. A similar study in 2019 assessed donor response when a legislation change was proposed in Belgium and concluded that only 20.1% of previous nonidentified donors would continue sperm donation if the law changed (24). In another analysis by Pennings et al. (33) in 2021, 53.8% of nonidentified donors surveyed stated they would stop donating if their anonymity was removed. However, when the law changed in Sweden in 1985, the country saw only a temporary dip in donor numbers, followed by a sharp increase (14). Similar findings were noted in Australia and the United Kingdom in 2005, as both countries saw an immediate increase in total sperm donors (26, 34). The United Kingdom has even seen a slight increase in the number of donors with more diverse ethnic backgrounds since this legislation change (28).

Although donor numbers do not seem to be affected by changing regulations on gamete donation, it is important to consider the few who still value their anonymity or who prefer no future contact in this process. Our sample showed that donors who originally made nonidentified donations were less comfortable with genetic data sharing than those who made open-identity donations and that open-identity donors were significantly more likely to donate again than nonidentified donors. The unexpected arrival of DTC-GT as an accessible identification resource can therefore have a detrimental psychosocial effect on donors and recipients who value their privacy. Thus, many donors and recipient families are calling for more counseling and support to manage the repercussions of contact or to prepare donors for contact from potential DCP (5).

Recipients, donors, and health care workers should be educated on the limits of anonymity in the setting of third-party reproduction. There has already been a shift toward psychoeducational counseling in the last decade, albeit with variable implementation across the country (35). The onus of education and support currently seems to fall mostly on the donors themselves (18). Donors should preferably receive implication counseling by qualified third-party reproduction professionals before donation, and those who are not willing to have future contact should be carefully informed of the contact risk before gamete donation. Donors should first be counseled on the implication of participating in DTC-GT, as it makes contact and linkage with DCP possible once testing occurs. Second, donors should be counseled on the possibility of DCP contact. Psychological, educational, and counseling resources must be developed, as donors may be far removed from their bank or clinic’s resources for managing DCP requests if contacted long after their donation. Donors should receive guidance on ways to talk with their families, partners, and children about their past and/or future donations. Third, resources for families of donors should be created to help these individuals navigate any future contact.

### Strengths and Limitations

This study addresses the important research gap on the influence of DTC-GT on donor experience in sperm donation. With the collaboration of 2 large national sperm banks, our survey yielded 396 responses from donors over the last 40 years. This multibank study on the novel topic of DTC-GT and its effects on gamete donation accumulated a large amount of demographic and donation history, as well as opinions and comments from the donors themselves.

A significant limitation to acknowledge is the 18.2% overall response rate of delivered surveys, which restricts the generalizability of the study. To maintain respondent anonymity, the survey was distributed via email by the respective sperm banks using contact information maintained by each bank. Email may not be the most effective method of communication between banks and their donors, leading to a lower response rate in our study. Many emails were not current or were invalid, which also reduced the pool of potential respondents.

It is also important to highlight that most respondents were recent donors who may not have been contacted by DCP from donations made between 2010 and 2020. Many respondents made open-identity donations, which could have led to more positive responses to DTC-GT. Finally, most donors have not participated in DTC-GT. Future research that includes a larger sample of donor attitudes toward DTC-GT and willingness to be contacted by DCP stratified by socioeconomic backgrounds may lead to more varied and insightful responses.

## Conclusion

This study sought to gather sperm donor opinions on DTC-GT services from the last 40 years in the United States now that DTC-GT is so widely accessible. Our findings show that donors are being contacted by DCP who identified them through DTC-GT, indicating that their company databases are making anonymity in the setting of third-party reproduction increasingly obsolete. Despite the increasing awareness of the possibility of lifelong contact from DCP through DTC-GT, many donors state they would donate again. As DTC-GT databases continue to grow, there is value in sperm donors receiving implication counseling from qualified third-party reproduction professionals before donation to consider future linkage to DCP, as well as ongoing counseling and support as donors navigate contact with DCP and establish these new types of relationships.
