# Supplementary material for: Sperm donor attitudes and experiences with direct-to-consumer genetic testing
Source: F S Rep. 2023 Feb 24;4(1):36–42. doi: 10.1016/j.xfre.2022.12.004 (PMC10028474; doi:10.1016/j.xfre.2022.12.004)
Supplement: Supplemental Material [file mmc1.docx]

Supplemental Material: Survey distributed to sperm donors

**Donor Demographics**

1. How would you describe yourself?

- American Indian or Alaska Native
- Asian
- Black or African American
- Hawaii Native or Other Pacific Islander
- Hispanic
- White
- Other __________________________________________________

1. What is your highest level of education?

- High school
- Undergraduate degree (college)
- Graduate degree (Masters)
- Professional degree (MD, JD, PhD, etc.)
- Other __________________________________________________

1. How would you describe your religious affiliation?

- Roman Catholic
- Protestant
- Muslim
- Jewish
- Spiritual but no specific religion
- Nonreligious
- Other __________________________________________________

1. What was your age at the time of your first sperm donation?
2. What was your age at the time of your last sperm donation?
3. What year(s) did you donate your sperm? (Check all that apply.)

| - 1980 - 1981 - 1982 - 1983 - 1984 - 1985 - 1986 - 1987 - 1988 | - 1989 - 1990 - 1991 - 1992 - 1993 - 1994 - 1995 - 1996 - 1997 | - 1998 - 1999 - 2000 - 2001 - 2002 - 2003 - 2004 - 2005 - 2006 | - 2007 - 2008 - 2009 - 2010 - 2011 - 2012 - 2013 - 2014 - 2015 | - 2016 - 2017 - 2018 - 2019 - 2020 |
| --- | --- | --- | --- | --- |

1. Which sperm bank did you donate to? (Check all that apply.)

- California Cryobank (CCB)
- Fairfax Cryobank
- Other __________________________________________________

1. What donor type did you choose at your time of donation? (Check all that apply.)

- Anonymous with no option of contact or further information exchange
- Open (agreed to participate in at least one facilitated contact with offspring once they are age 18)
- ID Disclosure (sperm bank to provide my identifying information to offspring once they are age 18)
- Other __________________________________________________

1. What was your original motivation for sperm donation? (Check all that apply.)

- Altruism (helping other couples conceive)
- To test my semen and overall fertility
- Financial compensation
- Personal satisfaction
- Other __________________________________________________

**Donor Social Information**

1. Do you currently have a partner?

- Yes
- No

*Display This Question:*

- *If “Do you currently have a partner?” = Yes*

1. Have you told your current partner about your sperm donation(s)?

- Yes
- No
- Unsure / Do not remember

1. Did you have a partner at the time of your donation(s)?

- Yes
- No
- Both partnered and non-partnered throughout my time as a donor

*Display This Question:*

- *If “Did you have a partner at the time of your donation(s)?” = Yes*
- *Or “Did you have a partner at the time of your donation(s)?” = Both partnered and non-partnered throughout my time as a donor*

1. Did you tell your partner about your sperm donation(s) at the time of your donation?

- Yes
- No
- Unsure / Do not remember

1. Do you currently have children?

- Yes, my own genetic children
- Yes, but not my genetic children (ie. stepchildren, adopted children, foster children)
- No

1. Did you have children at the time of your first donation?

- Yes, my own genetic children
- Yes, but not my genetic children (ie. stepchildren, adopted children, foster children)
- No

*Display This Question:*

- *If “Did you have children at the time of your first donation?” = No*

1. Did you have children throughout your time as a donor?

- Yes, my own genetic children
- Yes, but not my genetic children (ie. stepchildren, adopted children, foster children)
- No

1. Have you been open with the following people about your donation(s)?

|  | Yes | No | Unsure | N/A |
| --- | --- | --- | --- | --- |
| Your family |  |  |  |  |
| Your children |  |  |  |  |
| Your friends |  |  |  |  |
| Your colleagues |  |  |  |  |
| Members of your community (clergy, etc.) |  |  |  |  |

**Consumer Genetic Testing Information**

1. Have you participated in direct-to-consumer genetic testing (ie. 23andMe, Ancestry.com, etc.)?

- Yes
- No

*Display This Question:*

- *If “Have you participated in direct-to-consumer genetic testing (ie. 23andMe, Ancestry.com, etc.)?” = Yes*

1. Which company? (Check all that apply.)

- 23andMe
- Ancestry.com
- Other __________________________________________________

*Display This Question:*

- *If “Have you participated in direct-to-consumer genetic testing (ie. 23andMe, Ancestry.com, etc.)?” = Yes*

1. When did you participate in direct-to-consumer genetic testing? (Check all that apply.)

- Prior to my sperm donation
- During my time as a sperm donor
- After my last donation

*Display This Question:*

- *If “Have you participated in direct-to-consumer genetic testing (ie. 23andMe, Ancestry.com, etc.)?” = Yes*

1. What were your reasons for participating in direct-to-consumer genetic testing? (Check all that apply.)

- To learn more about my health
- To learn more about my ancestry
- General curiosity
- To discover genetically related people
- To make myself identifiable to any donor conceived offspring
- Other __________________________________________________

*Display This Question:*

- *If “Have you participated in direct-to-consumer genetic testing (ie. 23andMe, Ancestry.com, etc.)?” = No*

1. What were your reasons for not participating in direct-to-consumer genetic testing? (Check all that apply.)

- I did not want myself identifiable to any potential donor conceived offspring
- I did not feel comfortable having my genetic information stored by a third party
- I do not want to know what genetic conditions I may carry
- I am not curious about my ancestry
- Other __________________________________________________

1. Have any of your family members participated in direct-to-consumer genetic testing?

- Yes
- No
- Unsure

**Contact Information**

1. As a sperm donor, how comfortable are you that companies such as 23andMe and Ancestry.com are sharing the identity of genetically related persons?

| Extremely comfortable | Somewhat comfortable | Neither comfortable nor uncomfortable | Somewhat uncomfortable | Extremely uncomfortable |
| --- | --- | --- | --- | --- |
|  |  |  |  |  |

1. Are you open to contact with your donor conceived offspring? (Check all that apply.)

- Never
- Yes, at any age
- Yes, at least elementary school age
- Yes, at least middle school age
- Yes, at least high school age
- Yes, at age 18
- Undecided

*Display This Question:*

- *If “Are you open to contact with your donor conceived offspring?” = Yes, at any age; Yes, at least elementary school age; Yes, at least middle school age; Yes, at least high school age; Yes, at age 18*

1. Do you expect the sperm bank to moderate the contact between yourself and your donor conceived offspring?

- Yes
- No
- Unsure / Do not know

1. Are you open to contact with the family of your donor conceived offspring? (Check all that apply.)

- Never
- Yes, at any age
- Yes, at least elementary school age
- Yes, at least middle school age
- Yes, at least high school age
- Yes, at age 18
- Undecided

*Display This Question:*

- *If “Are you open to contact with the family of your donor conceived offspring?” = Yes, at any age; Yes, at least elementary school age; Yes, at least middle school age; Yes, at least high school age; Yes, at age 18*

1. Do you expect the sperm bank to moderate the contact between yourself and the family of your donor conceived offspring?

- Yes
- No
- Unsure / Do not know

*Display This Question:*

- *If “Are you open to contact with your donor conceived offspring?” = Yes, at any age; Yes, at least elementary school age; Yes, at least middle school age; Yes, at least high school age; Yes, at age 18*
- *Or “Are you open to contact with the family of your donor conceived offspring?” = Yes, at any age; Yes, at least elementary school age; Yes, at least middle school age; Yes, at least high school age; Yes, at age 18*

1. What is your primary reason for being open to contact from donor conceived offspring or their family members? (Check all that apply.)

- To establish a relationship with the offspring
- To provide the offspring with genetic or family history
- Curiosity
- A sense of obligation/right thing to do
- Other __________________________________________________

1. Did knowing the results of your direct-to-consumer genetic testing influence your attitude towards being contacted by donor conceived offspring?

| Not at all | Negatively influenced | Somewhat negatively influenced | Somewhat positively influenced | Positively influenced | N/A |
| --- | --- | --- | --- | --- | --- |
|  |  |  |  |  |  |

1. Have you been directly contacted by offspring conceived with your donated sperm?

- Yes
- No

*Display This Question:*

- *If “Have you been directly contacted by offspring conceived with your donated sperm?” = Yes*

1. How many offspring have contacted you?

*Display This Question:*

- *If “Have you been directly contacted by offspring conceived with your donated sperm?” = Yes*

1. Who contacted you? (Check all that apply.)

- Donor conceived offspring
- Parents of donor conceived offspring
- Other family member of donor conceived offspring
- Other ________________________________________________

1. Has anyone in your family been contacted by any of the following? (Check all that apply.)

- None
- Donor conceived offspring
- Parents of donor conceived offspring
- Other family member of donor conceived offspring
- Other __________________________________________________

*Display This Question:*

- *If “Have you been directly contacted by offspring conceived with your donated sperm?” = Yes*
- *Or “Has anyone in your family been contacted by any of the following?” = Donor conceived offspring; Parents of donor conceived offspring; Other family member of donor conceived offspring; Other*

1. How did the person who contacted you find your identity? (Check all that apply.)

- Direct to consumer DNA testing (ie. 23andMe, Ancestry.com)
- Through the donor sibling registry site
- Social media
- Through information search groups or individuals
- Through official/professional services (ie. private detective/specialist)
- Unsure / Do not know
- Other __________________________________________________

*Display This Question:*

- *If “Have you been directly contacted by offspring conceived with your donated sperm?” = Yes*
- *Or “Has anyone in your family been contacted by any of the following?” = Donor conceived offspring; Parents of donor conceived offspring; Other family member of donor conceived offspring; Other*

1. How would you describe your experience of being contacted by donor conceived offspring or their families?

| Very positive | Positive | Mixed | Negative | Very negative |
| --- | --- | --- | --- | --- |
|  |  |  |  |  |

*Display This Question:*

- *If “Have you been directly contacted by offspring conceived with your donated sperm?” = Yes*
- *Or “Has anyone in your family been contacted by any of the following?” = Donor conceived offspring; Parents of donor conceived offspring; Other family member of donor conceived offspring; Other*

1. Please share comments on your experience of being contacted by donor conceived offspring or their family members.
2. What are your opinions on sperm donation now that direct to consumer genetic testing (ie. 23andMe, Ancestry. com) is widely available to the public?
3. Would you feel comfortable donating sperm again knowing that companies such as 23andMe and Ancestry.com are sharing the identity of genetically related persons?

- Yes
- No
- Unsure / Do not know
